# Applying a Multilayer Construct of Social Adaptability Skills Within Talent Development

**DOI:** 10.3389/fpsyg.2019.03006

**Published:** 2020-01-15

**Authors:** Samuel Owiti, Denis Hauw, David Collins

**Affiliations:** ^1^Department of Sports Science, Institut des Sciences du Sport de l'Université de Lausanne, Lausanne, Switzerland; ^2^Moray House School of Education and Sport, University of Edinburgh, Edinburgh, Scotland; ^3^Grey Matters Performance Ltd., Stratford-upon-Avon, United Kingdom

**Keywords:** personality, narratives, experience, situated approach, club transition, talent development

## Introduction

Talent identification and development (TID) places emphasis on the ability to achieve at the highest level, focusing on a range of variables including adaptations to the context in which talent is embedded (Collins et al., 2018; Hauw, 2018). This adaptation affects the relationship to social constraints such as the variation in club cultures, coaches' style, teammates behavior, family displacement, and geographical constraints (Pulakos et al., 2000). Adaptive abilities will play a key role in optimizing returns from the biopsychosocially complex challenges inherent in any Talent Development Environment (TDE), whether formal (e.g., sport-specific academies) or in earlier, informal settings (Collins et al., 2012). Despite this evidence, an individual's Social Adaptability Skills (SAS) and their appropriate deployment are overlooked in TID models (Vaeyens et al., 2008).

Reflecting this importance, the aim of this article is to provide a theoretical model that encompasses the various psychological processes included in (SAS). Our reflection is guided first by the overview of the limits and resources of the current constructs accepted in TID and TDE's. We suggest the need for an integrative model combined in the SAS with the continuity of the athlete's identity as well as the integration of the inevitable situated and dynamical fluctuations. We subsequently examine the relevance of McAdams (2009) framework by inductively exploring meaningful testimonies of elite players transitions.

## What Psychological Skills Are Currently Seen as Needed on the Pathway?

To date, several constructs have been suggested as key for effective negotiation of the talent pathway and a few differences have been identified in this required skill set between formal or informal (e.g., club settings) TDEs, with most work focusing on general mental skills or attitudes rather than domain-specifics. The generic skills often mentioned in the literature, Growth Mindset (GM—Dweck, 2017) and Perseverance or specifically GRIT (Duckworth et al., 2007), can be seen broadly as attitudes which the developing performer brings to the sport. Elements that may or may not have been applied to other elements of his or her life. Both models have been criticized on the basis of methodology, psychometric validity, or application (e.g., Credé et al., 2017), although these models have substantial popularity and “street cred” in educational and developmental circles.

The other two approaches, resilience and self-regulation, can be seen as skills; methods which, once learned, can be applied to meet specific challenges along the pathway (Toering and Jordet, 2015; Fletcher and Sarkar, 2016). Of the two, only self-regulation seems to have the inherent genericity to adapt to social challenges such as the ones we raise in this paper (cf. Toering and Jordet, 2015). We posit that it is important to distinguish between adaptability as an outcome and the processes that produce it. Strategies such as coping are not, on their own, enough to explain how individuals manage environmental constraints. Secades et al. (2016) argue that coping is a *part* process (*appraisal of stressor, meta-cognition in response to emotions, and selection of strategy*) which only *could* lead to an outcome of adaptability.

In considering how exactly the skills needed are effectively achieved and developed, Collins et al. (2018) presented the P-O-P Model to address the challenges encountered by the psychosocial skills. This Model distinguished between Performance (desired target), Outcome (methods), and Processes (the means of achieving the outcomes leading to the desired performance). The Psychological Characteristics of Developing Excellence (PCDEs—MacNamara et al., 2010) were proposed as a skillset which can effectively meet these process requirements. However, all these approaches may have overlooked the importance of the psychosocial setting and how well the developing performer can adapt to and exploit it.

## Social Adaptability—A Missing Skill?

To date, research in sports has only emphasized the flexible and adaptive nature of athlete-environment interactions during skill acquisition and performance (e.g., Davids et al., 2013). No existing studies have either formulated methods or established structures and tools to measure SAS in sports. We therefore suggest some directions for further research, starting with an interrogation of SAS in athletes who succeed.

At a general level, SAS includes creative adaptations to coaches' expectations, coaching styles, coaches, and teammates from diverse cultural backgrounds, career transitions, geographical constraints, and being away from family. An appropriate ratio between “*stability*” (i.e., persistent behaviors), and “*flexibility*” (i.e., variable behaviors) might be consistent here inspiring from *motoric* behavior (e.g., Savelsbergh and Wormhoudt, 2019). The particularity of adaptability is the capacity for this ratio also to be varied (i.e., produce behavior that is stable or flexible as needed), as suggested by the example of a talented American player who joined a European team and was forced to adapt to the new environment.

“*In America, we are used to an individualistic play, but this was a big challenge when I came to Europe, where it's a collective type of game. I adapted well in my first year but…, I wasn't a team player, and I'm still learning. I can bring my winning mentality like when I was in the USA and score a lot of points, and my team would just follow”* (Owiti and Hauw, in review).

Here, the player found both the coaching and playing styles very challenging and admitted to adaptability concerns. Stability existed in his former team where he was comfortable and could perform well. While flexibility existed in his new team, when forced to adapt to the new environment, behavior change was embedded and at the same time resistant to perturbations. In this regard, *stability* and *flexibility* should not be construed as opposites on a continuum. Notably, *flexibility* would be not a loss of *stability* but, conversely, a sign of *adaptability*.

## The Relevance of McAdams (2009) for SAS

**Figure 1:**
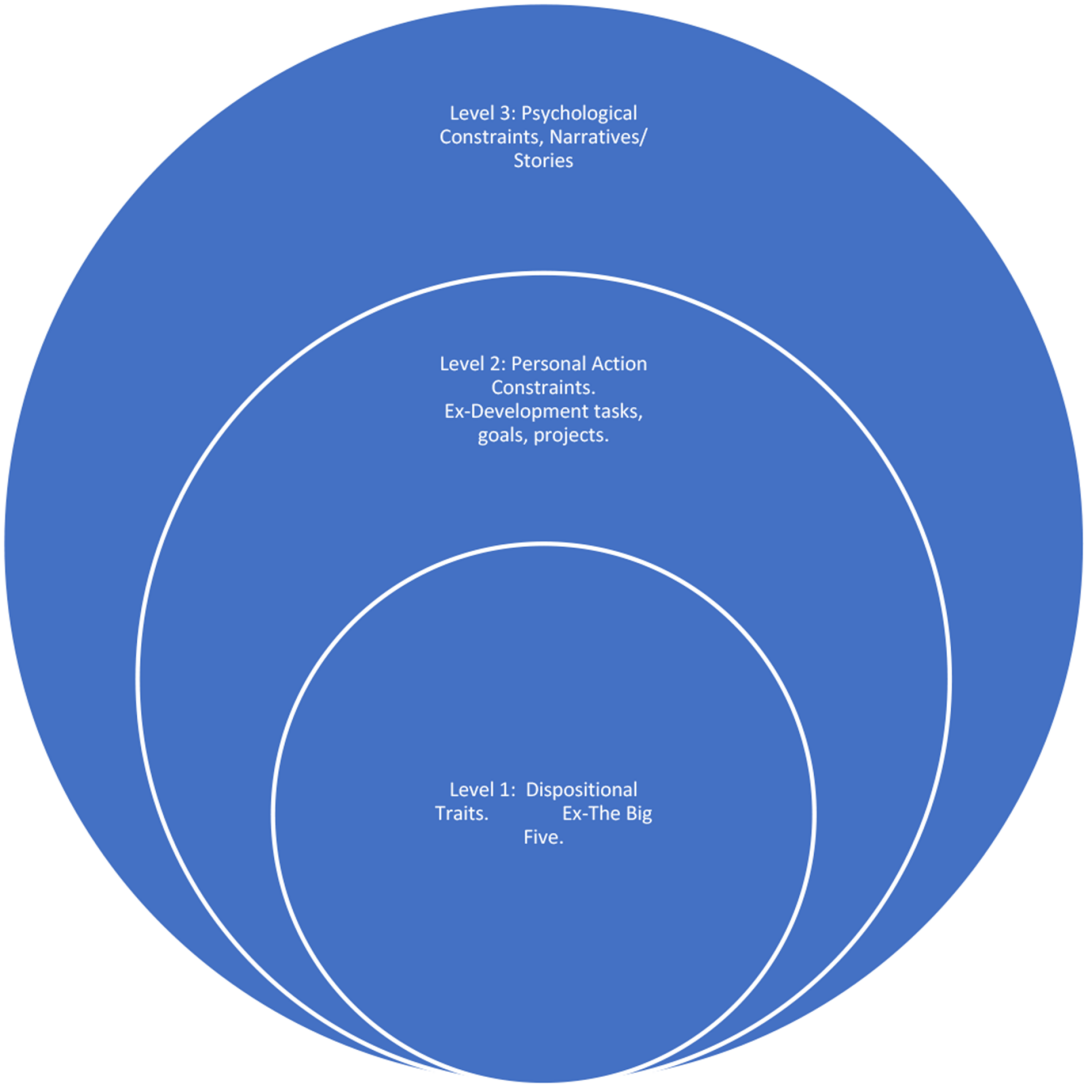
Mcadams identity multi-layer model (McAdams, 2009).

At a specific level, the Identity Multilayer Model (McAdams, 2009) would promote a fruitful, relevant understanding, and cater for the practical considerations of the SAS in the context of TID. McAdams (2009) indicates that identity can be defined through three layers of understanding (see Figure 1), incorporating (i) dispositional traits, (ii) Personal Action Constructs (PAC's), and (iii) Narratives.

Layer one comprises broad general personality traits (e.g., *Openness to experience, Extraversion, Conscientiousness, Emotional stability, and Agreeableness*) (Costa and McCrae, 2010) and is illustrated in the following:

*Boris Diaw, a former French NBA player, encountered difficult moments at Atlanta, Charlotte, and Utah Clubs. He had this to say about his time at Utah; “I put myself knowingly at the service of the collectiveness as a role of facilitator, I could not adapt to their individuality style. However, arriving at Spurs team, the coach allowed individuals to evolve together and also pull the best out of everyone.”* (Owiti and Hauw, in review).

Diaw had first to manage a complete long career in another country with a different culture. The need to adapt to different environments requires a set of mental dispositions or traits such as *openness to experience*. Dispositions toward others concerning *agreeableness* and *extraversion* also appear to be crucial here. At Utah, for example, the resources required to adapt to individualism were constrained while at the next transition, Diaw's dispositional traits facilitated adaptation to a collective type of play. It is also worth noting that, to capture the personality trait enabling adaptability; dispositional traits are limited and can only go this far (McAdams, 2009).

Layer two of McAdams model includes characteristic adaptations or PAC's that encompasses personal concerns, motivations, self-regulation or goal adaptations (Little, 2008); as illustrated in the following example:

“*… at times it can be difficult to adapt quickly, adaptations don't occur overnight, but you have to sacrifice your time, be mentally prepared, try to understand coach's style, set those short and long-term goals and once you put all pieces together, boom, puzzle solved”* (Owiti and Hauw, in review).

In this example, we see that this athlete used skills included in the PCDEQ portfolio to cope with the requirement (i.e., coping, motivation). This example also showed other skills, however, such as searching to understand the coach's expectations or considering short and long-term commitments. It is thus obvious that we need to more deeply explore what mental skills, attitudes and emotions refer to SAS and might be associated to the use of PCDEs within TID's.

The final level of McAdams model resides in stories constructed from experiences to give meaning to one's life (Bruner, 1990), as suggested in this example:

“*He was probably the toughest coach I ever had, his language, mannerisms and the tones he would use were unprofessional. He would yell and curse; it was crazy to experience; it was tough. That was probably the most challenging time for me as a player, and once I got through it, I knew that I would go through any challenge”* (Owiti and Hauw, in review).

This challenging transition with the coaching style grounded the personal experience of this player. The continuity of the identity of the player was progressively or suddenly fashioned with slow socialization or “nuclear episodes,” all under the “coherent theme” of the narrative identity. Therefore, this experience that was embedded, enacted and embodied could have helped the athlete in the adaptability process, because it could be re-injected throughout the whole career.

## Conclusion and Next Steps

Athletes' identity adaptation can be understood in terms of their dispositional traits as well as the regulations via their knowledge, goals, or values. However, to fully understand the adaptation, the integration of these two first levels, in addition to narratives that combine the past, present, and the future in personal experience all appear relevant. In sport, this multilayer perspective remains unexplored. Therefore, future studies could be deployed in two directions firstly, through a focus on each level of the model. For example: What are the traits, facets, and combinations required and how can we develop them in TDEs? What are the skills, knowledge and attitudes required to face uncertainty in this transition (PAC's) and how can they be assessed and developed? And lastly, at which dimensions and how can we generate a narrative experience that would be helpful for this transition in TDE and TID? As the second direction for study, research could focus on the dynamic relationship of these levels in retrospective and longitudinal studies in various sports.

## Ethics Statement

Informed consent was provided by the participants, with ethical declaration granted by the University Ethical Committee.

## Author Contributions

This article was written through the contribution of DH who gave great insights and ideas to it's conception. Further, DC provided his invaluable experience and ideas during the write up. Discussions were held regularly with the SO doing the write up, sending the drafts over to the other authors for comments.

### Conflict of Interest

DC was employed by Grey Matters Performance Ltd. The remaining authors declare that the research was conducted in the absence of any commercial or financial relationships that could be construed as a potential conflict of interest.
